# Force-based manipulation via wearable focal vibration for pain relief and changes in gait biomechanics in diabetic peripheral neuropathy: a secondary analysis of a pilot trial

**DOI:** 10.3389/fpain.2026.1925489

**Published:** 2026-08-10

**Authors:** Hongwu Wang, Raghuveer Chandrashekhar, Kyle W Ruffing, Roger B Fillingim

**Affiliations:** 1 Department of Occupational Therapy, Institute for Driving, Activity, Participation, and Technology, College of Public Health and Health Professions, University of Florida, Gainesville, FL, United States; 2 Human Engineering Research Laboratories, University of Pittsburgh, Pittsburgh, PA, United States; 3 Department of Neurology, College of Medicine, University of Florida, Gainesville, FL, United States; 4 College of Dentistry and the UF Pain Research & Intervention Center of Excellence, University of Florida, Gainesville, FL, United States

**Keywords:** diabetic peripheral neuropathy, force-based manipulation, gait biomechanics, neuropathic pain, neurophysiological regulation gait performance, focal vibration therapy

## Abstract

**Background:**

Diabetic peripheral neuropathy (DPN) disrupts somatosensory feedback, causing chronic pain, altered gait mechanics, and elevated fall risk. While force-based manipulations are known to modulate nociception and proprioception, their biomechanical effects and clinical efficacy in DPN remain underexplored. This secondary analysis investigated the effects of a wearable, force-based focal vibration therapy (FVT) on pain relief, estimated fall risk category, and gait performance in adults with DPN.

**Methods:**

We analyzed data from our single-blinded, parallel-group pilot trial of 28 participants (Experimental Group [EG], *n* = 25; Sham Control Group [CG], *n* = 3). Participants completed a 4-week home-based intervention. Clinical outcomes included the Timed Up-and-Go (TUG), Berg Balance Scale, and Brief Pain Inventory (BPI-DPN). Spatiotemporal, kinematic, and kinetic gait parameters were captured via 3D motion analysis. Given the small control sample, analyses prioritized within-group standardized effect sizes (Cohen's $d_{z}$) and clinical Minimal Clinically Important Difference (MCID) responder thresholds.

**Results:**

The force-based intervention yielded exceptional compliance. In the EG, FVT significantly reduced pain interference with walking (*p* = 0.015, $d_{z}$ =−0.54), and 44.4% of highly symptomatic participants achieved a clinically meaningful (≥3-point) reduction in worst pain. Functionally, continuous TUG times significantly improved (*p* = 0.033, $d_{z}$ =−0.93), resulting in 33.3% of high-risk patients successfully transitioning to a low fall-risk category (compared to a descriptive 0% in the CG). Biomechanically, these functional gains were accompanied by changes in gait biomechanics that may reflect improved gait performance, specifically, increased gait speed, reduced total double-support time (*p* = 0.014, $d_{z}$ =−0.53), and increased initial ground reaction forces, achieved without requiring compensatory proximal joint moments.

**Conclusion:**

Targeted force-based manipulation via wearable FVT offers a highly feasible mechanism for pain relief and changes in gait biomechanics in DPN. By uncoupling neuropathic pain from functional ambulation, this intervention provides preliminary, hypothesis-generating evidence that supports the need for further investigation in fully powered trials.

## 1 Background

Over time, people with diabetes develop nerve damage accompanied by well-documented debilitating symptoms (1). Up to 70% of people with diabetes develop neuropathies, with risk increasing both with age and disease duration (2). Diabetic peripheral neuropathy (DPN) is the most common type of peripheral neuropathy. It affects approximately 50% of all patients with diabetes and 1% of the world population (3–5). The estimated annual cost of diabetes in the U.S. in 2012 was $245 billion, and about 27% of the health-care costs associated with diabetes can be attributed to DPN (6).

DPN is a symmetrical, length-dependent sensorimotor polyneuropathy attributed to metabolic and microvascular alterations induced by hyperglycemia and concomitant cardiovascular risk factors. DPN affects the sensory, motor, and autonomic components of the nervous system, leading to a loss of protective sensation and intrinsic foot muscle dysfunction, which may alter gait (7). Research documents that patients with DPN exhibit postural instability and gait imbalance, which contribute to the incidence of falls, partially due to significant deficits in sensorimotor function and balance (8–10). Existing solutions for DPN-related gait instability include medication, diet control, mechanical aids, physical therapy, massage, acupuncture, and exercise, all with limitations regarding availability, side effects, and cost; factors that prevent widespread use and leave many patients with limited symptom relief (11–15).

In recent reviews of diabetic neuropathy and gait, Alam et al. (9) and Wang et al. (10) documented that gait abnormalities in patients with DPN are closely linked to alterations in kinetics, kinematics, and posture. Gait abnormalities may lead to an increased risk of falls and be a significant cause of morbidity and mortality in older people with diabetes (11). The authors further suggested that a detailed evaluation of gait disorders in DPN is vital, and research on sustainable interventions for DPN-related gait abnormalities is imperative (9). Aerobic and resistance exercise regimens for patients with diabetes can improve gait stability by enhancing muscle strength, increasing joint range of motion, and maintaining consistent management of blood sugar, blood pressure, and serum lipid levels (16–19). There was, however, a lack of consensus on the frequency and duration of program management as well as the sustainability of these effects. Patients with DPN also struggle with compliance when treatment does not occur in a clinical setting (20).

Crucially, the relationship between DPN-induced pain and reduced mobility is cyclical. While absolute pain intensity (e.g., worst or average pain) is a primary complaint, pain sub-domains, specifically how pain interferes with general activity and walking ability, are direct precursors to sedentary behavior, functional decline, and heightened fall risk (21). Therefore, interventions must not only address sensory deficits but also effectively alleviate pain interference with daily mobility to foster long-term functional independence.

Targeted mechanical force-based interventions, such as focal vibration therapy (FVT), have emerged as promising instrument-assisted methods to address these somatosensory disturbances. Previous studies have found that focal vibration reduces muscle spasticity (22–25) facilitates muscle contraction (26, 27), and stimulates the proprioceptive system to enhance efficient motor control during functional activities (28). Emerging research suggests that mechanical stimuli applied directly to the musculature can act as an endogenous pain modulator, potentially influencing peripheral and segmental neuronal sensitization (29). Indeed, our recent comprehensive systematic review evaluating 62 FVT trials confirmed that targeted mechanical stimulation of lower-extremity musculature, particularly the quadriceps, gastrocnemius/soleus, and plantar muscles, significantly optimizes gait propulsion and postural stability (30). Furthermore, this synthesis identified a critical neurophysiological therapeutic window: vibration frequencies between 80 and 120 Hz and amplitudes of 0.2–0.5 mm consistently maximize the activation of muscle spindle Ia afferents, thereby augmenting sensorimotor organization and proprioceptive feedback (30). Patients with multiple sclerosis, dystonia, stroke, and incomplete spinal cord injuries have demonstrated improvement in gait cadence, step length, and walking speed after consistent use of focal vibration (31–34).

We previously established the feasibility of a home-based wearable FVT intervention operating within this optimal high-frequency window and reported significant pre-post improvements in spatiotemporal gait parameters (35), as well as overall pain and mobility (36) within the active treatment cohorts. However, the initial reports (35, 36) did not evaluate these outcomes against the sham control arm, nor did they assess the clinical significance of the findings using Minimal Clinically Important Difference (MCID) responder thresholds, estimated fall risk category shifts, detailed individual kinematic/kinetic mechanisms, or specific pain-interference subcategories.

Furthermore, there remains a critical knowledge gap regarding how force-based manipulation influences the gait biomechanics for safe mobility in DPN. Therefore, this secondary analysis aims to evaluate the comparative effects of FVT versus sham on clinical endpoints in the complete pilot cohort of 28 participants (*n* = 25 allocated to active FVT modes; *n* = 3 allocated to sham control). Specifically, we aim to 1) evaluate the estimated fall risk category shifts in high fall-risk status using established mobility cut-offs, 2) determine the proportion of clinical pain responders achieving MCID, and 3) assess the targeted impact of FVT on alleviating pain interference with walking ability. By integrating clinical pain responder metrics with functional mobility outcomes, this analysis seeks to inform individualized manipulation strategies and clarify the therapeutic benefits of mechanical force in managing neuropathic pain.

## 2 Materials and methods

### 2.1 Study design

We conducted a secondary analysis of a single-site, single-blinded, parallel-group, randomized clinical trial. The clinical assessors administering the TUG and BBS, as well as the personnel conducting the post-intervention 3D gait analyses and processing the motion capture data, were blinded to the participants’ group allocation. While the primary trial focused on the feasibility of the home-based wearable FVT intervention and pre-post clinical measures of pain, balance, mobility, and gait, this secondary analysis focuses on the comparative effects against sham control, clinical responder thresholds, and exploratory mechanistic outcomes. It is important to note that the unbalanced randomization allocation (25 active treatment vs. 3 sham) was an intentional design choice for this initial pilot. The primary objective was not to perform a fully powered efficacy comparison against a control, but rather to conduct an exploratory parameter-selection analysis across three distinct vibration modes (pulsed, sinusoidal, and continuous). The aggregated active-treatment data provide the variance estimates necessary to power a future, balanced, randomized controlled trial. The study was approved by the University of Oklahoma Health Sciences Center (OUHSC) Institutional Review Board (#9688).

### 2.2 Participants

Participants were recruited and randomly assigned to one of three intervention groups or the control group after they gave their consent: Intervention group 1 received a pulsed on-and-off vibration at 120 Hz; Intervention group 2 received a sinusoidal vibration ranging between 35 and 120 Hz; Intervention group 3 received continuous vibration at a constant frequency of 120 Hz; and the control group received a sham vibration with a vibration sound but no real vibration. The Myovolt wearable vibration device offers these three modes, and sham vibration with sound only was used for this study. While a computer-generated randomization sequence was initially utilized, early drop-outs and scheduling conflicts resulted in uneven final allocations across the specific modes. Inclusion criteria for this study were: 1) diagnosis of diabetes for at least one year; 2) diabetic peripheral neuropathy as defined by failure to sense the 5.07 (10 g) monofilament test in one or more of the six sites tested (37); 3) age 18–75 years old; 4) able to ambulate independently without assistive devices (e.g., walker or crutches) for 30 feet; 5) no evidence of neurological (other than peripheral neuropathy) or orthopedic conditions; 6) able to understand English instructions; and 7) have normal or corrected vision. Individuals with other non-diabetic causes of neuropathy by history, symptomatic peripheral vascular disease, joint pain, swelling and/or limited of range of motion in the lower extremities that interfere with walking, other systemic or local diseases that could interfere with walking assessment, amputation in the lower extremities, clinically diagnosed with dementia greater than mild (screened using Montreal Cognitive Assessment (38) (MOCA) < 24) were excluded. A physician or therapist with relevant training in DPN care confirmed the eligibility of subjects. All subjects interested in the study were informed about the procedure and the research aim. Eligible and willing patients signed an institutional review board-approved informed consent prior to screening. All subjects were recruited from Oklahoma City and the surrounding area, with support from the Harold Hamm Diabetes Center.

#### 2.2.1 Sample size

Because no previous research had been conducted on the effect of focal vibration in patients with DPN, we performed power calculations for the primary pilot trial using MCID when available, along with reasonable estimates of expected change. We based our calculations on our primary outcomes: Timed-Up and Go (TUG), pain, and Berg Balance Scale (BBS). With an alpha of 0.05, a power of 0.8, and a paired t-test, we needed 8 subjects per group to detect within-subject changes. As this is a secondary analysis of the completed pilot cohort, no *a priori* power calculation was performed for the specific subgroup responder comparisons.

### 2.3 Outcome measures

The primary feasibility measures assessed in the parent trial include recruitment rates, retention rates, compliance, safety, and acceptability. We tracked the number of participants contacted, assessed for eligibility, and the number of eligible participants who agreed to participate and completed the enrollment process to calculate recruitment rates. Similarly, the number of participants who completed all required assessments was recorded and used to calculate retention rates. Compliance was calculated based on the Myovolt usage log prepared for each participant. Safety was monitored through documented adverse events associated with Myvolt use. Acceptability was evaluated using the assistive technology subscale of the Quebec User Evaluation of Satisfaction with Assistive Technology (QUEST 2.0) (39). The assistive technology subscale of QUEST 2.0 consists of 8 items, with a total possible score of 40, each scored on a scale of 1–5 (39).

The primary balance and mobility measures were assessed using the BBS (40), and the Cognitive and standard TUG (41) tests, respectively. We used the Brief Pain Inventory -Diabetic Peripheral Neuropathy (BPI-DPN) (42) for pain assessment.

We collected quantitative gait parameters using the Qualysis™ (Qualisys AB, Göteborg, Sweden) motion capture system and AMTI™ (Advanced Mechanical Technology, Inc., Watertown, MA, USA) force plates, placing reflective markers on 58 anatomical landmarks, using the Qualysis™ motion capture system's 12 cameras (sampling rate 120 Hz) and the AMTI™ force plates (sampling rate 1 kHz) to capture gait performance data. Each participant first performed a static trial to develop their individual skeletal model, which the authors then exported to Visual3D^©^ (C-Motion, Germantown, MD, USA) for data analysis. Participants then walked at a self-selected pace for 25 feet in three to five trials, using the pace they used in everyday life. We then calculated kinematic and kinetic data using Visual3D^©^. Those gait parameters were analyzed: gait speed, stride length, stride width, stride time, left and right stance time, left and right swing time, cadence, duration of double support, left and right peak knee flexion, left and right peak dorsiflexion, left and right peak plantarflexion, left and right peak knee flexor moment, left and right peak plantar flexor moment, and left and right peak ankle joint power. Visual3D^©^ defines the spatiotemporal measures as follows (43): gait speed is the stride length divided by stride time, stride length is the distance from heel strike to heel strike on the same side, stride time is the duration of the gait cycle from heel strike to heel strike on the same side, left stance time is the time from left heel strike to left toe off, right stance time is the time from right heel strike to right toe off, left swing time is the time from left toe off to left heel strike, right swing time is the time from right toe off to right heel strike, mean cadence is the average number of steps taken per minute, and duration of double support is the amount of time spent with both feet in contact with the surface.

### 2.4 Study protocol

Demographic data (age, weight, height, duration of type II diabetes mellitus, gender, and ethnicity) and baseline balance, mobility, pain, and gait data were collected at the first visit. The FVT intervention included a 4-week at-home program using a modified version of a commercially available wearable FVT device (Myovolt™, Christchurch, New Zealand), one for each leg. The modified version consisted of a single vibration motor with a recommended amplitude of 1.2 mm. The authors asked participants to wear the device over their typical clothing and use it as they preferred during their daily activities.

Participants wore the modified Myovolt wearable device on both legs and applied 10 min of vibration to the distal quadriceps muscle/tendon, the gastrocnemius/soleus muscle/tendon, and the tibialis anterior muscle group, with an intersession interval of 1 min each day, three days a week, for 4 weeks. Participants in the control group wore the sham Myovolt, which produced the same vibration sound but no vibration. The sham Myovolt appears identical to the experimental device. The authors trained all participants to wear, remove, and charge the Myovolt. A simple, intuitive user manual was provided to all participants.

We completed gait and balance measures at baseline and 4 weeks (end of the vibration therapy period). At the end of the study, we also collected feedback on the intervention. Preliminary studies on the acute and short-term benefits of focal vibration for gait and balance disturbances in other movement disorders used a 4-week intervention, so the comparison will be similar. Further, there needed to be sufficient time for each user to become comfortable and familiar with the device to achieve a therapeutic outcome.

#### 2.4.1 Study variables

*Demographics Characteristics*: These include age (date of birth); duration of injury (date of DPN); stage of DPN; and current medications, co-morbidities, and secondary conditions – including covariates that might limit or interfere with normal function. The Common Data Elements structure was used whenever appropriate (44).

*Gait Analysis*: We measured quantitative gait parameters at the Center for Human Performance Measurement (CHPM) using the Qualysis™ motion capture system and AMTI™ force plates. Each participant who met the selection criteria was tested during a single 3-hour session at baseline and at the end of 4 weeks. During these sessions, gait kinematic, kinetic, and spatiotemporal data were concurrently captured and assessed. Participants underwent gait testing at two speeds (self-paced and brisk-paced), which are feasible for preliminary work in other motion-analysis patients. The ground reaction force and gait data were sampled at 2 kHz. While both self-paced and brisk-paced testing were conducted, only the self-paced trials were analyzed for this manuscript to best reflect activities of daily living.

*Berg Balance Scale (BBS)*: An objective measure of balance. It has been used to identify and evaluate balance impairment. Previous studies have shown that the BBS is a valid (45) and reliable (40) measure of balance in patients with diabetes.

*Timed Up & Go*: The Timed Up and Go test is a mobility measure that requires both static and dynamic balance. Previous studies have validated the TUG in patients with DPN (41, 46).

*Utility of Myovolt Technology*: We administered the Quebec User Evaluation of Satisfaction with Assistive Technology (QUEST 2.0) (39) to assess users’ satisfaction with the Myovolt.

*Participant Logs*: We asked participants to complete logs recording the date, time, and frequency of device use, as well as problems encountered during free use. Study personnel monitored participants to identify any adverse events and assess adherence to study protocols.

*Experience and Impressions (Qualitative)*: We conducted semi-structured interviews with participants and, in some cases, family and/or caregivers to obtain their impressions of the device, the intervention, and its potential for success. Additionally, interview information helped us better understand how suitable the device is for this population of users.

### 2.5 Data and statistical analysis plan

All statistical analyses were performed using RStudio (version 2026.05.1). Due to the small sample size in the sham control group (*N* = 3), formal between-group inferential statistics were not conducted to avoid violating test assumptions. Instead, analyses focused on within-group changes for the active FVT cohort using paired t-tests (or Wilcoxon signed-rank tests for non-parametric data). Descriptive statistics and standardized effect sizes (Cohen's $d_{z}$) were calculated to demonstrate the magnitude of the intervention. Because this secondary analysis was strictly exploratory and aimed at generating hypotheses for future efficacy trials, formal adjustments for multiple comparisons were intentionally not applied to prioritize statistical sensitivity in detecting preliminary biomechanical changes. We also conducted a categorical risk analysis to assess clinical significance. Participants were dichotomized into ‘High Fall Risk’ (TUG >12 s) and ‘Low/Normal Fall Risk’ (TUG ≤ 12 s) at baseline. We evaluated the proportion of high-risk participants who successfully transitioned across this clinical threshold to a normal mobility status post-intervention.

Because the primary aim of the parent pilot study was feasibility, participants were not excluded based on low baseline pain scores. To account for potential floor effects in the BPI-DPN, a secondary responder and subgroup analysis were conducted. Participants were stratified by baseline pain severity. We defined clinical responders as those achieving an MCID of ≥3 points (47) in the Worst Pain domain. Descriptive statistics and median changes were calculated specifically for the subgroup experiencing severe baseline pain (≥ 7/10) to evaluate the intervention's potential in a clinically symptomatic population.

To provide a physiological rationale for the observed functional improvements in TUG and BBS, spatiotemporal and kinetic variables (e.g., peak ankle joint power, stride length) were analyzed as exploratory mechanistic outcomes. Rather than serving as primary endpoints, these biomechanical markers were analyzed to generate hypotheses regarding the neuromuscular mechanisms (e.g., improved proprioceptive feedback vs. increased muscle activation) driving clinical changes.

Qualitative analyses: To obtain the most comprehensive understanding of motivation, barriers, and a range of individual experiences, perceptions, and impressions, it was invaluable to collect this information through semi-structured interviews throughout the study. We developed exploratory approaches based on the content matter in these interviews.

## 3 Results

### 3.1 Participant flow and demographics

**Figure 1:**
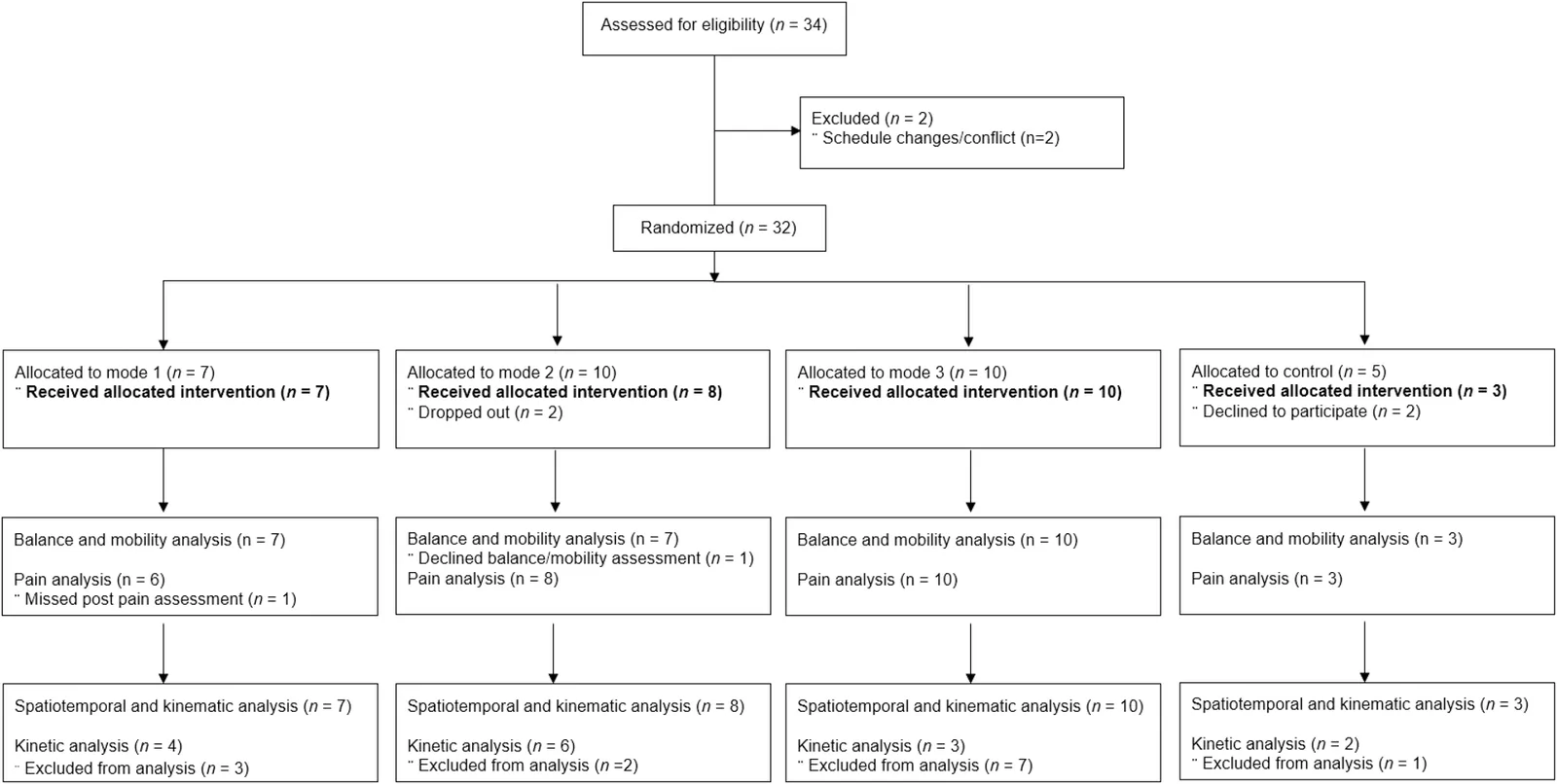
CONSORT chart.

A total of 34 individuals with diabetic peripheral neuropathy were assessed for eligibility; 32 were randomized to the study. Two participants in the sinusoidal intervention group dropped out after the baseline visit, and two participants in the sham control group declined to participate after trying the sham device due to a lack of perceived usefulness. The final analyzed cohort consisted of 28 participants (FVT active groups, *n* = 25; Sham control group, *n* = 3). For the purposes of this secondary analysis, the three active vibration modes were aggregated into a single Experimental Group (EG; *n* = 25), compared with the Sham Control Group (CG; *n* = 3). Within the EG, 24 participants completed the balance and mobility analysis, and 24 completed the pain analysis. Seven participants used an assistive device (a cane or walker) during data collection and were excluded from the kinetic data analysis, as shown in Figure 1, the CONSORT chart. Variations in sample sizes for specific kinetic and kinematic variables (e.g., *n* = 6–13) were due to marker occlusion or failure to achieve an isolated foot placement on the force plates during walking trials.

Baseline demographics (Table 1) showed no significant statistical differences in age (*p* = 0.99), weight (*p* = 0.42), height (*p* = 0.49), BMI (*p* = 0.37), or years with diabetes (*p* = 0.51) among the EG and CG.

**Table 1:** Participant demographics: age, weight, height, body mass index, and years of diabetes diagnosis are all listed as mean (standard deviation), while number, sex, and race are listed as *N*.

Demographics/group	All participants	Intervention group 1	Intervention group 2	Intervention group 3	Control group	*p*-Value*
Number	28	7	8	10	3	–
Age (years)	67.07 (9.96)	66.71 (13.35)	68.13 (5.82)	66.90 (12.08)	65.67 (4.73)	0.99
Weight (lb)	217.11 (55.95)	194.14 (44.21)	238.00 (53.95)	223.60 (63.11)	193.33 (60.28)	0.42
Height (in)	67.52 (3.58)	65.86 (3.90)	67.38 (4.21)	68.55 (2.77)	68.33 (3.79)	0.49
Body Mass Index (BMI) (lb/in2)	33.35 (7.88)	31.19 (5.28)	36.92 (7.56)	33.42 (9.60)	28.62 (6.28)	0.37
Years with Diabetes diagnosis	18.11 (9.02)	16.43 (5.56)	22.25 (13.71)	16.80 (6.78)	15.33 (6.43)	0.51
Sex (F/M)	17/11	5/2	4/4	6/4	2/1	–
Ethnicity						
Caucasian	23	6	8	9	0	–
African American	3	0	0	1	2	–
Eurasian	1	1	0	0	0	–
American Indian/Alaska Native	1	0	0	0	1	–

*Analysis of variance (ANOVA) results. Part of this table was published in our previous manuscript by Rippetoe et al. (35).

### 3.2 Feasibility, compliance, and satisfaction

The home-based wearable FVT intervention demonstrated high feasibility and adherence. For participants who completed the intervention, device usage compliance was excellent, with most participants achieving 100% of the recommended usage days (Table 2). Overall satisfaction with the device was high across all modes, with total satisfaction scores consistently ranging from 31 to 40 out of 40 (Table 3). Furthermore, preliminary qualitative feedback from semi-structured interviews aligned with these high satisfaction scores, with participants frequently noting the device's ease of use and comfort during daily activities, supporting its feasibility for independent home use. No adverse events were reported related to the use of the FVT or sham device.

**Table 2:** Satisfaction scores and compliance.

Pt. ID.	Mode	Days used	Recommended use	Compliance days or sessions used/recommended use	Total satisfaction with device
1	3	12	12	100	24
2	3	12	12	100	31
3	3	12	12	100	38
4	3	12	12	100	38
5	3	12	12	100	35
6	3	NR	12	NR	24
7	2	12	12	100	40
8	2	12	12	100	40
9	2	12	12	100	38
10	1	12	12	100	34
11	1	12	12	100	32
12	1	NR	12	NR	34
14	1	12	12	100	40
15	2	12	12	100	33
16	3	12	12	100	38
17	1	12	12	100	34.6
21	2	12	12	100	37
22	3	36	36	100	32
23	1	34	36	94.4	NR
24	1	24	24	100	39
26	3	24	24	100	17
27	3	12	12	100	38
28	2	12	12	100	40
29	2	28	33	84.8	38
30	2	12	12	100	36
31	4	12	12	100	32
32	4	12	12	100	40
34	4	12	12	100	38

NR means not reported. Values are missing because the participant either failed to return their device usage log at the end of the 4-week intervention or left the satisfaction questionnaire incomplete.

**Table 3:** Summary of QUEST 2.0 satisfaction scores by mode.

Group	Mode	*N* ^a^	Mean satisfaction score (Max 40)	Standard deviation (SD)
Intervention Group 1	Pulsed	6	35.60	3.16
Intervention Group 2	Sinusoidal	8	37.75	2.43
Intervention Group 3	Continuous (120 Hz)	10	31.50	7.49
Control Group	Sham	3	36.67	4.16

a One participant in Group 1 did not complete the satisfaction questionnaire.

### 3.3 Clinical mobility and estimated fall risk category transition

Following the 4-week intervention, participants in EG demonstrated significant, large-magnitude within-group improvements in dynamic balance and mobility (Table 4). Descriptive evaluation indicated that the CG did not exhibit similar therapeutic trends.

**Table 4:** Pain, balance, mobility, and sensation scores before and after a 4-week focal muscle vibration (FMV) intervention for all participants.

Outcome measure	*N*	Time of measurement	Mean (SD)	95% confidence interval (of mean or median of ranks)	*p*-Value
BPI-DPN	EG – 24	Pre	3.41 (2.55)	(−0.04, 0.79)	0.07*
		Post	2.87 (1.92)		
	CG – 2	Pre	1.73 (2.44)	N/A	N/A
		Post	5.00 (1.54)		
BBS^a^	EG – 24	Pre	41.88 (10.12)	(0.00, 4.50)	0.068
		Post	44.21 (9.69)		
	CG – 3	Pre	50.67 (3.51)	N/A	N/A
		Post	51.00 (3.61)		
TUG^a^	EG – 24	Pre	13.92 (5.33)	(−2.24, −0.05)	0.033*
		Post	12.69 (5.09)		
	CG – 3	Pre	12.18 (4.07)	N/A	N/A
		Post	13.01 (4.46)		
TUG cognitive	EG – 23	Pre	15.58 (7.25)	(−4.29, −1.07)	0.002*
		Post	12.89 (5.55)		
	CG – 2	Pre	14.41 (7.46)	N/A	N/A
		Post	14.54 (7.39)		
SWMT Right foot^a^	EG – 25	Pre	4.92 (3.34)	(−0.50, 1.00)	0.430
		Post	5.24 (3.22)		
	CG – 3	Pre	8.00 (1.00)	N/A	N/A
		Post	7.67 (4.04)		
SWMT Left foot^a^	EG – 25	Pre	4.64 (3.29)	(−0.50, 1.50)	0.160
		Post	5.24 (3.59)		
	CG – 3	Pre	7.00 (4.36)	N/A	N/A
		Post	7.67 (3.21)		

a means data is not normally distributed, and the Wilcoxon Signed Rank Test was used.

*means *p* < 0.05 and the result is statistically significant; BPI-DPN is Brief Pain Inventory–Diabetic Peripheral Neuropathy; BBS is Berg Balance Scale; TUG is standard Timed Up-and-Go; TUG cognitive is the cognitive Timed Up-and-Go; SWMT is Semmes–Weinstein Monofilament Test; *N* is number of participants analyzed; BPI-DPN scores are measured out of 10 and the scores are scaled to a score between 0 and 10. Mean ± SD has been reported for all outcomes in the CG group, but the sample size was insufficient for inferential testing.

Continuous TUG Metrics: The EG exhibited a statistically significant decrease in standard TUG completion time from 13.92 ± 5.33 s at baseline to 12.69 ± 5.09 s post-intervention (*p* = 0.033). This improvement represents a large within-group standardized effect size (Cohen's $d_{z}$  = −0.93 [95% CI: −2.24, −0.05]). Similar moderate to large-magnitude improvements were observed under dual-task conditions, with the EG demonstrating a substantial within-group reduction in Cognitive TUG times (Cohen's $d_{z}$  = −0.77 [95% CI: −4.29, −1.07]). In descriptive contrast, the CG showed worsening completion times across both standard and cognitive mobility metrics over the 4-week period.

Vibration Mode Comparisons: Visual analysis (Supplementary Figure A1 in Appendix) of estimated marginal means indicated that all three active modes (pulsed, sinusoidal, and continuous) reduced TUG and Cognitive TUG times, but the continuous 120 Hz vibration mode yielded the lowest absolute completion times post-intervention.

Categorical Fall Risk (Responder Analysis): While continuous variables reveal overall cohort trends, a categorical fall risk analysis was conducted to assess the intervention's functional clinical significance. At baseline, 60.7% (*n* = 17) of the cohort had a standard TUG completion time>12.0 s, indicating high fall risk. Following the 4-week intervention, 33.3% of the high-risk participants in the EG (*n* = 5/15) improved their mobility times sufficiently to cross this clinical threshold into the normal/low fall-risk category (≤12.0 s). In contrast, 0% of the high-risk participants in the CG (*n* = 0/2) achieved this transition (Risk Difference = 33.3%).

### 3.4 Neuropathic pain relief and sub-domain interference

Because participants were not excluded based on mild baseline pain, the overall cohort mean for baseline pain was relatively low (3.32/10; see Table 4), indicating a statistical floor effect. Despite this, analyzing specific BPI-DPN sub-domains (Table 5) revealed highly targeted benefits of the mechanical force intervention within the EG.

**Table 5:** Item scores on BPI–DPN pre- and post-intervention for all participants.

Outcome measure	*N*	Time of measurement	Mean (SD)	95% confidence interval (of mean or median of ranks)	*p*-Value
Worst Pain	EG – 24	Pre	4.42 (3.44)	(−0.34, 0.46)	0.758
		Post	4.25 (3.15)		
	CG – 2	Pre	4.00 (5.66)	N/A	N/A
		Post	5.00 (2.83)		
Least Pain	EG – 24	Pre	2.50 (2.92)	(−0.17, 0.64)	0.252
		Post	1.92 (1.61)		
	CG – 2	Pre	1.00 (1.41)	N/A	N/A
		Post	4.50 (3.54)		
Average Pain	EG – 24	Pre	3.79 (2.34)	(−0.11, −0.97)	0.014*
		Post	3.08 (2.26)		
	CG – 2	Pre	3.00 (4.24)	N/A	N/A
		Post	6.00 (1.41)		
Current Pain	EG – 24	Pre	2.75 (2.74)	(−0.15, 0.66)	0.220
		Post	2.13 (1.65)		
	CG – 2	Pre	2.00 (2.83)	N/A	N/A
		Post	4.50 (3.54)		
General Activity	EG – 24	Pre	3.00 (3.31)	(−0.12, 0.70)	0.164
		Post	2.25 (1.94)		
	CG – 2	Pre	0.00 (0.00)	N/A	N/A
		Post	4.00 (1.41)		
Mood	EG – 24	Pre	2.71 (2.94)	(−0.56, 0.25)	0.457
		Post	3.04 (2.84)		
	CG – 2	Pre	2.50 (3.54)	N/A	N/A
		Post	3.50 (0.71)		
Walking Ability^a^	EG – 24	Pre	4.08 (3.23)	(−0.10, −0.96)	0.015*
		Post	3.04 (2.03)		
	CG – 2	Pre	0.00 (0.00)	N/A	N/A
		Post	5.00 (4.24)		
Interference to Normal Walking	EG – 24	Pre	3.63 (2.95)	(−0.20, 0.61)	0.322
		Post	3.08 (2.55)		
	CG – 2	Pre	0.00 (0.00)	N/A	N/A
		Post	5.50 (0.71)		
Relationships	EG – 24	Pre	2.04 (2.84)	(−0.36, 0.45)	0.824
		Post	1.96 (2.22)		
	CG – 2	Pre	0.00 (0.00)	N/A	N/A
		Post	3.50 (2.12)		
Sleep	EG – 24	Pre	4.71 (3.88)	(−0.11, 0.71)	0.151
		Post	4.08 (3.39)		
	CG – 2	Pre	3.50 (4.95)	N/A	N/A
		Post	10.00 (0)		
Enjoyment	EG – 24	Pre	3.83 (3.75)	(−0.19, 0.60)	0.298
		Post	3.25 (2.52)		
	CG – 2	Pre	3.00 (4.24)	N/A	N/A
		Post	3.50 (2.52)		

a means data is not normally distributed, and the Wilcoxon Signed Rank Test was used.

*means *p* < 0.05 and the result is statistically significant; N is the number of participants analyzed. Mean ± SD has been reported for all outcomes in the CG group, but the sample size was insufficient for inferential testing.

**Figure 2:**
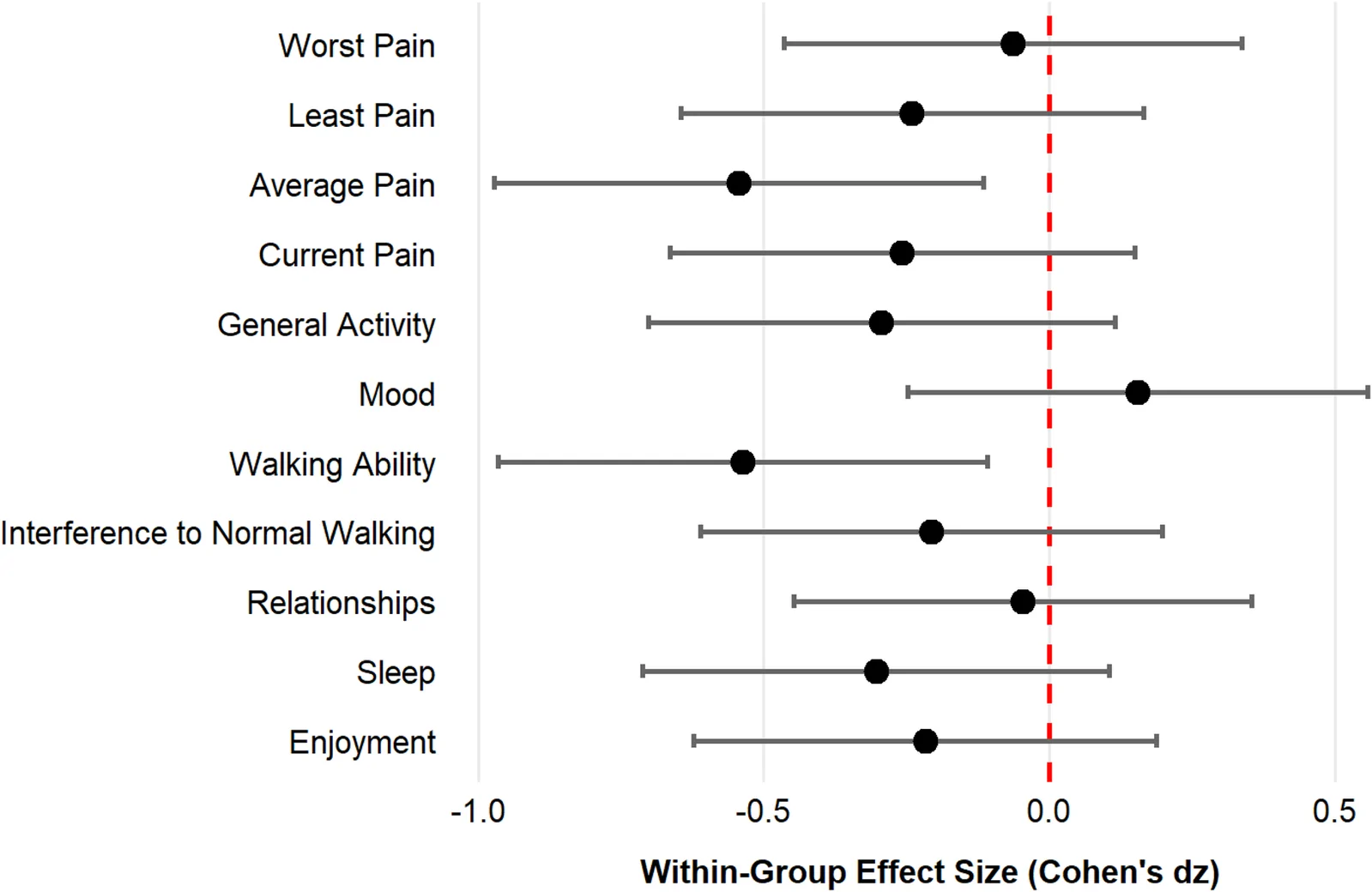
Standardized within-group effect sizes (Cohen's $d_{z}$) of FVT across BPI-DPN sub-domains for the EG. Conventional thresholds for effect size magnitude are defined as small (*d*_z_ = 0.2), medium (*d*_z_ = 0.5), and large (*d*_z_ = 0.8). Effect sizes are derived from pre-to-post intervention changes, represented as mean Cohen's $d_{z}$ with 95% confidence intervals (error bars). Values < 0 indicate a clinical improvement (reduction in pain intensity or interference) from baseline, while values > 0 indicate a worsening of symptoms. The vertical dashed red line represents the null hypothesis of no change ($d_{z}$ = 0). The EG demonstrated consistent, directionally favorable reductions across key interference domains, with statistically significant, moderate-to-large therapeutic effects observed specifically in Average Pain ($d_{z}$ = −0.54 [95% CI: −0.97, −0.11]) and Walking Ability ($d_{z}$ = −0.54 [95% CI: −0.96, −0.10]).

Pain Intensity: The EG experienced a statistically significant within-group reduction in ‘Average Pain’ scores (*p* = 0.014), yielding a moderate, standardized effect size (Cohen's $d_{z}$  = −0.54 [95% CI: −0.97, −0.11], Figure 2). In descriptive contrast, the CG showed a descriptive worsening in average pain over the 4-week period.

Pain Interference with Mobility: Highlighting the functional mechanism of the therapy, FVT significantly reduced the extent to which neuropathic pain interfered with ‘Walking Ability’ within the EG (*p* = 0.015). This improvement represented a moderate within-group effect size (Cohen's $d_{z}$  = −0.54 [95% CI: −0.96, −0.10], Figure 2). Descriptively comparing this to the CG, which experienced descriptive worsening in this exact domain, suggests that FVT effectively uncoupled neuropathic pain from functional ambulation, facilitating the clinical mobility improvements observed in the TUG fall-risk analysis.

MCID Subgroup Analysis: To directly address the cohort floor effect, a targeted responder analysis was conducted for the subset of patients who entered the trial with severe ‘Worst Pain’ (≥ 7/10). In this clinically symptomatic EG subgroup (*n* = 8), participants achieved a mean intra-individual pain reduction of 2.38 points, demonstrating a large within-group effect (Cohen's $d_{z}$  = 1.05 [95% CI: 0.48, 4.27]). Clinically, 44.4% of these highly symptomatic participants in the active treatment group met the established MCID of ≥ 3-point absolute reduction in worst pain.

### 3.5 Biomechanical gait analysis

Comprehensive pre- and post-intervention biomechanical gait data for all participants are provided in Supplementary Appendix Tables A1–A3. Due to the high attrition rate and small sample size of the CG (*n* = 3) for spatiotemporal/kinematics; *n* = 1–2 for kinetics), inferential statistics were computed exclusively to evaluate within-group changes for the EG.

#### 3.5.1 Spatiotemporal gait parameters

Following the 4-week FVT intervention, the EG demonstrated statistically significant improvements across several key spatiotemporal parameters. Participants exhibited a highly significant increase in overall gait speed (*p* < 0.001, $d_{z}$  = 0.82 [95% CI: 0.05, 0.14]) and a significant increase in stride length (*p* = 0.010, $d_{z}$  = 0.56 [95% CI: 0.01, 0.09]). Overall cycle time was significantly reduced (*p* = 0.001, $d_{z}$  = −0.78 [95% CI: −0.15, −0.04]). This was driven by significant decreases in stance times for both the left (*p* = 0.006, $d_{z}$  = −0.61 [95% CI: −0.11, −0.02]) and right (*p* = 0.013, $d_{z}$  = −0.53 [95% CI: −0.13, −0.02]) limbs. Crucially, total double support time, a primary indicator of gait instability, was significantly reduced post-intervention (*p* = 0.014, $d_{z}$  = −0.53 [95% CI: −0.10, −0.01]), alongside significant reductions in bilateral initial double support phases (*p* < 0.05). Stride width remained unchanged (*p* = 0.878).

#### 3.5.2 Kinematic variables

Kinematic analysis revealed stable joint geometries across most phases of the gait cycle, with one localized significant change. The EG demonstrated a significant increase in Right Hip Angle during Maximum Stance, increasing from 22.28° to 27.49° (*p* = 0.046, $d_{z}$  = 0.42 [95% CI: 0.09, 10.32]). Maximum and minimum joint angles of the bilateral ankles and knees across both stance and swing phases did not change significantly from baseline (all *p* > 0.05).

#### 3.5.3 Kinetic variables

Analysis of ground reaction forces (GRF), joint moments, and joint powers revealed a targeted increase in ground engagement with stable joint kinetics. The baseline mean of 0.00 for Right GRF at Heel Strike reflects the adoption of a compensatory flat-foot landing strategy by these participants, wherein the force plate threshold for a distinct, isolated heel-strike peak was not triggered prior to the intervention. The EG exhibited a highly significant increase in Right GRF at Heel Strike (*p* = 0.001, $d_{z}$  = 2.17 [95% CI: 0.17, 0.42]) and a significant increase in Left GRF at Toe-Off (*p* = 0.041, $d_{z}$  = 0.98 [95% CI: 0.01, 0.32]). Bilateral ankle, knee, and hip moments and powers across maximum/minimum stance and swing phases remained stable.

## 4 Discussion

This pilot, randomized, sham-controlled trial investigated the preliminary clinical efficacy and underlying biomechanical mechanisms of a 4-week home-based FVT in adults with DPN. The results demonstrate that wearable FVT is a highly feasible and well-tolerated non-pharmacological intervention. Furthermore, the 4-week application of FVT drove significant, large-magnitude improvements in clinical mobility, reduced estimated fall risk category, and drastically diminished neuropathic pain interference in highly symptomatic patients. Crucially, comprehensive gait analysis revealed that these clinical improvements are underpinned by specific spatiotemporal and kinetic optimizations, suggesting that FVT is associated with changes in gait biomechanics that could reflect improved gait performance. This shift in estimated fall risk category underscores that FVT does not merely yield statistically significant reductions in continuous time but functionally reclassifies a third of highly vulnerable patients out of a high fall-risk status. Given the exploratory nature of this secondary analysis, we did not apply statistical corrections for multiple comparisons. Therefore, all statistically significant findings, particularly those with marginal *p*-values (e.g., Right Hip Angle, *p* = 0.046), should be interpreted strictly as hypothesis-generating rather than confirmatory.

### 4.1 Feasibility and adherence in a home-based paradigm

Current pharmacological treatments for DPN are often limited by incomplete efficacy, dose-limiting side effects, and poor long-term adherence (48). In this study, the home-based wearable FVT intervention demonstrated exceptional feasibility. Device usage compliance was excellent, with most participants in the active cohort achieving 100% of the recommended usage days, and overall satisfaction was high. The success of this remote, self-administered model validates FVT's scalability. By utilizing interactive training and a “teach-back” method, the intervention effectively circumvents the logistical and health literacy barriers that frequently limit the adoption of clinic-bound rehabilitation technologies (49).

### 4.2 Clinical mobility and estimated fall risk category transition

DPN heavily degrades distal somatosensory feedback, leading to altered gait mechanics and a disproportionately high fall risk (8–10). Following the intervention, the active FVT group exhibited significant, large-magnitude reductions in continuous TUG and Cognitive TUG completion times (Cohen's $d_{z}$  = −0.93 and −0.77, respectively). However, the most clinically meaningful finding is the shift in estimated fall risk category. At baseline, many participants exhibited TUG times >12.0 s, a well-established threshold for high fall risk in older adults (50). Post-intervention, 33.3% of high-risk participants in the active FVT group successfully transitioned to the low fall-risk category (≤12.0 s), compared with 0% in the sham control group. This functional reclassification demonstrates that FVT yields outcomes that are not only statistically significant but clinically transformative for patient safety and independence.

### 4.3 Neuropathic pain relief and sub-domain interference

Evaluating pain strictly through whole-cohort averages can obscure targeted therapeutic effects, particularly when populations include participants with mild baseline symptoms. While the overall cohort exhibited a 1-point reduction in pain, a targeted responder analysis was utilized to assess the subset of patients with severe baseline pain (Worst Pain ≥ 7/10). In this symptomatic subgroup, 44.4% of the active FVT group achieved a ≥ 3-point absolute reduction in worst pain, exceeding the established MCID for the Brief Pain Inventory in DPN populations (47). Beyond absolute intensity, FVT produced systemic, moderate-magnitude improvements in pain interference. The intervention uncoupled neuropathic pain from functional ambulation and restorative rest, yielding moderate standardized effect sizes for improvements in Walking Ability Interference ($d_{z}$ = −0.54) and Average Pain ($d_{z}$ = −0.54). Contrastingly, the sham control group descriptively showed descriptive deterioration across these exact domains, indicating that the absence of active mechanical force allowed the underlying neuropathic interference to progress unabated.

### 4.4 Exploratory biomechanical changes and gait performance

The significant reductions in estimated fall risk category and neuropathic pain interference observed in this cohort were underpinned by profound, systematic alterations in gait biomechanics. DPN typically degrades distal proprioceptive and cutaneous feedback, forcing patients to adopt a cautious, hesitant gait strategy characterized by prolonged double-support time, shortened strides, and reduced joint excursion (8–10, 51). The targeted application of FVT effectively reversed these compensatory mechanisms, suggesting that the mechanical force intervention may be associated with changes in gait strategy (52). When interpreting these biomechanical findings, it is crucial to distinguish between measured gait performance and hypothesized physiological changes. Because double-support time is strongly influenced by overall functional capacity, these findings could equally represent improved gait performance driven by increased walking velocity and reduced pain, rather than a definitively altered locomotor strategy. The observed changes may hypothetically relate to increased movement confidence.

Following the 4-week FVT intervention, the experimental group demonstrated a significant transition toward a more physiological gait pattern. Participants exhibited highly significant increases in gait speed and stride length, paired with a concurrent reduction in overall cycle time. Most notably, the intervention led to a significant decrease in both left and right stance times, resulting in a robust reduction in total double-support time (*p* = 0.014). In neuropathic populations, prolonged double-support is a primary compensatory strategy utilized to maximize ground contact when ascending sensory information is unreliable. The significant reduction in double-support time may reflect augmented afferent sensory signaling, though it is also highly consistent with increased patient confidence, reduced pain interference, and the mathematical dependency on increased gait speed (53). It must be noted that improvements in variables such as stride length, cycle time, and stance duration are inherently mathematically coupled to the significant increase in overall gait speed. Future studies utilizing speed-matched or normalized analyses are required to isolate independent kinematic adaptations from velocity-driven changes.

Kinematically, the experimental cohort exhibited localized optimizations, most notably a significant increase in right hip extension during maximum stance (*p* = 0.046), which indicates improved trailing-limb posture (54). This optimized hip kinematics actively tensions the anterior hip capsule and flexor musculature, storing elastic energy essential for the subsequent swing phase.

Kinetically, the joint kinetic parameter, specifically lower limb moments and power, remained largely stable across the intervention. However, targeted improvements were observed in ground reaction forces (GRF), with significant increases at right heel strike (*p* = 0.001) and left toe-off (*p* = 0.041). The FVT group's ability to significantly increase gait speed and stride length without proportionally larger joint moments suggests a profound improvement in overall motor efficiency (55, 56). Rather than relying on compensatory, energy-costly proximal muscle contractions (57), a hallmark of neuropathic gait (9), patients achieved a faster, more stable gait through improved spatiotemporal timing and more confident ground engagement. While increased GRF indicates more robust ground engagement, this metric is heavily influenced by increased walking velocity and improved forward propulsion, rather than serving as direct proof of enhanced proprioception (58). Therefore, it must be acknowledged that increases in ground reaction force may simply reflect increased walking velocity and greater propulsive demand, independent of any actual changes in sensory function or gait strategy. Ultimately, these biomechanical shifts confirm that FVT provides more than palliative pain relief. By utilizing a targeted mechanical force to upregulate ascending somatosensory feedback, the intervention fundamentally reorganizes the kinematic and kinetic integrity of the gait cycle, directly mitigating the mechanical mechanisms that precipitate high fall risk in DPN.

In conclusion, this study advances the paradigm of non-pharmacological DPN management. By translating clinic-based focal vibration into a feasible, wearable home-based technology, this intervention offers a scalable solution that may potentially address biomechanical and sensorimotor deficits that drive fall risk in diabetic populations.

### 4.5 Limitations and future directions

While this pilot study provides compelling preliminary evidence for the efficacy of home-based FVT, several limitations must be noted. These limitations have been critical in refining the methodology for subsequent, fully powered efficacy trials. The primary limitation of this study is the small overall sample size (*N* = 28) and the unequal final group distribution, resulting in an active treatment group of 25 participants and a sham control group of only 3 participants. This imbalance precluded the use of formal between-group inferential statistics for pain, clinical mobility, and biomechanical outcomes. While the within-group effect sizes for the active FVT group were moderate and directionally consistent (e.g., Cohen's $d_{z}$  > 0.5 for primary pain interference domains), the restricted CG sample limits the generalizability of these findings. This underscores the necessity of a future double-blinded, balanced, and fully powered randomized controlled trial to rigorously confirm these effects against a robust sham condition. Second, including participants with mild baseline pain resulted in a statistical floor effect in the overall cohort. When viewed as an overall average, the cohort experienced a 1-point reduction in pain and a 1-second reduction in the standard TUG. While statistically significant, a 1-point reduction in pain does not meet the established 3-point MCID for the BPI-DPN. This was driven by a statistical floor effect, as participants with mild baseline pain were not excluded from the pilot. By addressing this limitation through targeted responder analyses, we demonstrated that the subgroup of participants with severe baseline pain successfully achieved the ≥3-point MCID, and high-risk patients successfully transitioned below the critical 12-second TUG fall-risk cut-off. Future efficacy studies must utilize these specific severity thresholds in their inclusion criteria to avoid floor effects and ensure that clinically meaningful functional outcomes remain the primary endpoint. Furthermore, given the exploratory nature of this secondary analysis, we intentionally did not apply statistical corrections for multiple comparisons (e.g., Bonferroni or FDR). In small pilot cohorts, conservative adjustments can inflate the risk of Type II errors (false negatives), leading to the premature dismissal of potentially valuable clinical changes. Our statistical approach prioritized sensitivity to identify biomechanical trends and generate targeted hypotheses for future, fully powered efficacy trials. Therefore, all statistically significant findings, particularly those with marginal *p*-values (e.g., Right Hip Angle, *p* = 0.046), should be interpreted strictly as hypothesis-generating rather than confirmatory. Finally, the exploratory nature of this pilot necessitated the collection of a vast array of physiological, biomechanical, and psychosocial assessments to identify potential mechanisms of action. While this broad approach successfully captured a large dataset, it yielded many secondary measures, limiting our ability to establish specific, directional *a priori* hypotheses. However, this limitation served its intended purpose: it allowed us to filter out low-yield variables and isolate the most sensitive mechanistic mediators (e.g., stance time, initial ground reaction forces, and mobility-specific pain interference). These refined variables may directly inform a highly specified, hypothesis-driven mechanistic aim in future trials, minimizing participant burden while precisely mapping the neurophysiological pathways of mechanical force therapy.

## Data Availability

The original contributions presented in the study are included in the article/Supplementary Material, further inquiries can be directed to the corresponding author.
