# Supplementary material for: Force-based manipulation via wearable focal vibration for pain relief and changes in gait biomechanics in diabetic peripheral neuropathy: a secondary analysis of a pilot trial
Source: Front Pain Res (Lausanne). 2026 Aug 10;7:1925489. doi: 10.3389/fpain.2026.1925489 (PMC13500243; doi:10.3389/fpain.2026.1925489)
Supplement: Supplementary file 1 [file Table1.docx]

**Appendix**

**Appendix Figure A1**. Estimated Marginal Means of Clinical and Mobility Outcomes across Vibration Modes. Visual representation of the estimated marginal means (EMM) for four primary clinical outcomes: Standard TUG (seconds), Cognitive TUG (seconds), Berg Balance Scale (0–56), and Average Pain Intensity (0–10). Data are presented as mean values with error bars representing standard error (SE). Solid colored lines represent the three active focal vibration therapy (FVT) modes (Pulsed, Sinusoidal, and Continuous 120 Hz), while the dashed gray line represents the Sham Control Group. Across all mobility and pain metrics, the Continuous 120 Hz vibration mode demonstrated the most consistent therapeutic trajectory, yielding the lowest absolute mobility completion times and pain scores, and the highest balance scores post-intervention

| **Appendix Table A1.** Spatiotemporal Gait Variables (Pre- and Post-Intervention) | | | | | | |
| --- | --- | --- | --- | --- | --- | --- |
| **Outcome Measure** | **Time** | **Group (N)** | **Mean (SD)** | **EG 95% CI** | **EG p-Value** | **EG Effect Size (d)** |
| **Gait Speed (m/s)** | Pre | EG (25) | 0.79 (0.32) | (0.05, 0.14) | **<0.001*** | 0.82 |
|  | Post |  | 0.88 (0.33) |  |  |  |
|  | Pre | CG (3) | 0.74 (0.26) |  |  |  |
|  | Post |  | 0.84 (0.25) |  |  |  |
| **Stride Length (m)** | Pre | EG (25) | 0.97 (0.28) | (0.01, 0.09) | **0.010*** | 0.56 |
|  | Post |  | 1.02 (0.27) |  |  |  |
|  | Pre | CG (3) | 0.93 (0.20) |  |  |  |
|  | Post |  | 1.01 (0.22) |  |  |  |
| **Stride Width (m)** | Pre | EG (25) | 0.18 (0.04) | (-0.01, 0.01) | 0.878 | 0.03 |
|  | Post |  | 0.19 (0.04) |  |  |  |
|  | Pre | CG (3) | 0.19 (0.00) |  |  |  |
|  | Post |  | 0.17 (0.02) |  |  |  |
| **Cycle Time (s)** | Pre | EG (25) | 1.33 (0.32) | (-0.15, -0.04) | **0.001*** | -0.78 |
|  | Post |  | 1.23 (0.26) |  |  |  |
|  | Pre | CG (3) | 1.32 (0.24) |  |  |  |
|  | Post |  | 1.23 (0.13) |  |  |  |
| **Left Step Length (m)** | Pre | EG (25) | 0.49 (0.13) | (-0.02, 0.05) | 0.369 | 0.18 |
|  | Post |  | 0.51 (0.13) |  |  |  |
|  | Pre | CG (3) | 0.47 (0.13) |  |  |  |
|  | Post |  | 0.51 (0.12) |  |  |  |
| **Right Step Length (m)** | Pre | EG (25) | 0.47 (0.16) | (0.02, 0.06) | **0.001*** | 0.74 |
|  | Post |  | 0.51 (0.15) |  |  |  |
|  | Pre | CG (3) | 0.46 (0.07) |  |  |  |
|  | Post |  | 0.50 (0.10) |  |  |  |
| **Left Step Time (s)** | Pre | EG (25) | 0.68 (0.20) | (-0.09, -0.02) | **0.002*** | -0.69 |
|  | Post |  | 0.62 (0.15) |  |  |  |
|  | Pre | CG (3) | 0.66 (0.08) |  |  |  |
|  | Post |  | 0.61 (0.05) |  |  |  |
| **Right Step Time (s)** | Pre | EG (25) | 0.65 (0.14) | (-0.07, -0.01) | **0.005*** | -0.61 |
|  | Post |  | 0.61 (0.12) |  |  |  |
|  | Pre | CG (3) | 0.66 (0.15) |  |  |  |
|  | Post |  | 0.62 (0.08) |  |  |  |
| **Left Stance Time (s)** | Pre | EG (25) | 0.89 (0.26) | (-0.11, -0.02) | **0.006*** | -0.61 |
|  | Post |  | 0.83 (0.22) |  |  |  |
|  | Pre | CG (3) | 0.89 (0.24) |  |  |  |
|  | Post |  | 0.82 (0.13) |  |  |  |
| **Right Stance Time (s)** | Pre | EG (25) | 0.90 (0.30) | (-0.13, -0.02) | **0.013*** | -0.53 |
|  | Post |  | 0.83 (0.22) |  |  |  |
|  | Pre | CG (3) | 0.90 (0.20) |  |  |  |
|  | Post |  | 0.82 (0.14) |  |  |  |
| **Left Swing Time (s)** | Pre | EG (25) | 0.42 (0.05) | (-0.03, -0.01) | **0.009*** | -0.57 |
|  | Post |  | 0.40 (0.05) |  |  |  |
|  | Pre | CG (3) | 0.44 (0.02) |  |  |  |
|  | Post |  | 0.41 (0.02) |  |  |  |
| **Right Swing Time (s)** | Pre | EG (25) | 0.42 (0.07) | (-0.04, 0.00) | 0.092 | -0.35 |
|  | Post |  | 0.41 (0.05) |  |  |  |
|  | Pre | CG (3) | 0.42 (0.02) |  |  |  |
|  | Post |  | 0.41 (0.01) |  |  |  |
| **Left Init. DBL Support** | Pre | EG (25) | 0.24 (0.11) | (-0.05, -0.01) | **0.008*** | -0.57 |
|  | Post |  | 0.21 (0.09) |  |  |  |
|  | Pre | CG (3) | 0.24 (0.12) |  |  |  |
|  | Post |  | 0.21 (0.09) |  |  |  |
| **Right Init. DBL Support** | Pre | EG (25) | 0.25 (0.15) | (-0.05, -0.00) | **0.041*** | -0.43 |
|  | Post |  | 0.22 (0.11) |  |  |  |
|  | Pre | CG (3) | 0.23 (0.09) |  |  |  |
|  | Post |  | 0.20 (0.06) |  |  |  |
| **Total DBL Support (s)** | Pre | EG (25) | 0.48 (0.25) | (-0.10, -0.01) | **0.014*** | -0.53 |
|  | Post |  | 0.42 (0.20) |  |  |  |
|  | Pre | CG (3) | 0.46 (0.22) |  |  |  |
|  | Post |  | 0.41 (0.14) |  |  |  |

*Note: EG = Experimental Group (FVT); CG = Control Group (Sham). The p-value, 95% CI, and Effect Size (Cohen's d_z_) represent the within-group, pre-to-post-intervention change calculated exclusively for the EG.*

| **Appendix Table A2.** Kinematic Gait Variables (Pre- and Post-Intervention) | | | | | | |
| --- | --- | --- | --- | --- | --- | --- |
| **Outcome Measure** | **Time** | **Group (N)** | **Mean (SD)** | **EG 95% CI** | **EG p-Value** | **EG Effect Size (d)** |
| **L Ankle Angle Max Stance** | Pre | EG (25) | 20.11 (4.79) | (-1.24, 1.68) | 0.756 | 0.06 |
|  | Post |  | 20.33 (5.53) |  |  |  |
|  | Pre | CG (3) | 19.47 (3.47) |  |  |  |
|  | Post |  | 16.91 (4.56) |  |  |  |
| **R Ankle Angle Max Stance** | Pre | EG (25) | 18.29 (7.58) | (-2.49, 4.39) | 0.573 | 0.11 |
|  | Post |  | 19.24 (3.09) |  |  |  |
|  | Pre | CG (3) | 4.60 (20.10) |  |  |  |
|  | Post |  | 16.56 (6.64) |  |  |  |
| **L Ankle Angle Max Swing** | Pre | EG (25) | 9.97 (6.04) | (-0.76, 3.21) | 0.216 | 0.25 |
|  | Post |  | 11.20 (8.76) |  |  |  |
|  | Pre | CG (3) | 9.68 (4.63) |  |  |  |
|  | Post |  | 6.60 (7.16) |  |  |  |
| **R Ankle Angle Max Swing** | Pre | EG (25) | 7.66 (6.53) | (-1.77, 5.07) | 0.330 | 0.20 |
|  | Post |  | 9.31 (4.81) |  |  |  |
|  | Pre | CG (3) | -5.64 (19.98) |  |  |  |
|  | Post |  | 5.08 (6.29) |  |  |  |
| **L Ankle Angle Min Stance** | Pre | EG (25) | -5.07 (6.27) | (-2.45, 2.71) | 0.918 | 0.02 |
|  | Post |  | -4.94 (7.46) |  |  |  |
|  | Pre | CG (3) | -0.98 (0.61) |  |  |  |
|  | Post |  | -10.22 (10.79) |  |  |  |
| **R Ankle Angle Min Stance** | Pre | EG (25) | -6.69 (8.46) | (-3.75, 5.20) | 0.742 | 0.07 |
|  | Post |  | -5.97 (6.89) |  |  |  |
|  | Pre | CG (3) | -18.89 (19.76) |  |  |  |
|  | Post |  | -6.49 (7.11) |  |  |  |
| **L Ankle Angle Min Swing** | Pre | EG (25) | -7.49 (8.50) | (-4.10, 2.00) | 0.484 | -0.14 |
|  | Post |  | -8.54 (9.58) |  |  |  |
|  | Pre | CG (3) | -1.84 (5.29) |  |  |  |
|  | Post |  | -13.35 (17.35) |  |  |  |
| **R Ankle Angle Min Swing** | Pre | EG (25) | -9.21 (9.67) | (-4.61, 4.93) | 0.945 | 0.01 |
|  | Post |  | -9.05 (9.12) |  |  |  |
|  | Pre | CG (3) | -19.42 (21.12) |  |  |  |
|  | Post |  | -9.38 (8.36) |  |  |  |
| **L Hip Angle Max Stance** | Pre | EG (25) | 23.46 (12.48) | (-2.00, 8.07) | 0.225 | 0.25 |
|  | Post |  | 26.50 (11.54) |  |  |  |
|  | Pre | CG (3) | 31.04 (2.93) |  |  |  |
|  | Post |  | 32.70 (6.06) |  |  |  |
| **R Hip Angle Max Stance** | Pre | EG (25) | 22.28 (12.54) | (0.09, 10.32) | **0.046*** | 0.42 |
|  | Post |  | 27.49 (12.80) |  |  |  |
|  | Pre | CG (3) | 29.31 (0.98) |  |  |  |
|  | Post |  | 31.18 (7.39) |  |  |  |
| **L Hip Angle Max Swing** | Pre | EG (25) | 26.21 (12.28) | (-2.30, 7.72) | 0.276 | 0.22 |
|  | Post |  | 28.92 (10.95) |  |  |  |
|  | Pre | CG (3) | 32.92 (3.77) |  |  |  |
|  | Post |  | 36.16 (4.28) |  |  |  |
| **R Hip Angle Max Swing** | Pre | EG (25) | 25.84 (12.85) | (-1.01, 9.39) | 0.110 | 0.33 |
|  | Post |  | 30.03 (13.02) |  |  |  |
|  | Pre | CG (3) | 30.97 (1.80) |  |  |  |
|  | Post |  | 33.19 (4.94) |  |  |  |
| **L Hip Angle Min Stance** | Pre | EG (25) | -8.24 (11.39) | (-3.77, 6.01) | 0.641 | 0.09 |
|  | Post |  | -7.12 (11.94) |  |  |  |
|  | Pre | CG (3) | -0.21 (7.57) |  |  |  |
|  | Post |  | 1.13 (7.55) |  |  |  |
| **R Hip Angle Min Stance** | Pre | EG (25) | -10.51 (9.60) | (-1.63, 8.48) | 0.174 | 0.28 |
|  | Post |  | -7.08 (10.84) |  |  |  |
|  | Pre | CG (3) | -1.41 (6.99) |  |  |  |
|  | Post |  | -0.14 (8.82) |  |  |  |
| **L Hip Angle Min Swing** | Pre | EG (25) | 1.70 (12.45) | (-3.86, 6.75) | 0.578 | 0.11 |
|  | Post |  | 3.15 (12.45) |  |  |  |
|  | Pre | CG (3) | 7.62 (6.21) |  |  |  |
|  | Post |  | 9.46 (5.73) |  |  |  |
| **R Hip Angle Min Swing** | Pre | EG (23) | 0.22 (10.97) | (-3.47, 7.60) | 0.447 | 0.16 |
|  | Post |  | 2.29 (12.22) |  |  |  |
|  | Pre | CG (3) | 8.09 (3.82) |  |  |  |
|  | Post |  | 9.76 (8.12) |  |  |  |
| **L Knee Angle at HS** | Pre | EG (25) | 4.33 (10.17) | (-1.64, 6.45) | 0.231 | 0.25 |
|  | Post |  | 6.74 (9.77) |  |  |  |
|  | Pre | CG (3) | 8.83 (5.27) |  |  |  |
|  | Post |  | 3.20 (6.76) |  |  |  |
| **R Knee Angle at HS** | Pre | EG (25) | 4.48 (10.66) | (-1.50, 10.18) | 0.138 | 0.31 |
|  | Post |  | 8.82 (10.78) |  |  |  |
|  | Pre | CG (3) | 2.27 (3.02) |  |  |  |
|  | Post |  | 2.15 (11.56) |  |  |  |
| **L Knee Angle Max Stance** | Pre | EG (25) | 38.62 (6.92) | (-1.04, 6.59) | 0.147 | 0.30 |
|  | Post |  | 41.39 (8.48) |  |  |  |
|  | Pre | CG (3) | 42.09 (7.08) |  |  |  |
|  | Post |  | 35.83 (1.20) |  |  |  |
| **R Knee Angle Max Stance** | Pre | EG (25) | 34.62 (11.36) | (-0.45, 10.66) | 0.070 | 0.38 |
|  | Post |  | 39.73 (10.40) |  |  |  |
|  | Pre | CG (3) | 40.65 (5.97) |  |  |  |
|  | Post |  | 41.13 (0.83) |  |  |  |
| **L Knee Angle Max Swing** | Pre | EG (25) | 51.85 (9.45) | (-0.69, 7.67) | 0.098 | 0.34 |
|  | Post |  | 55.34 (9.79) |  |  |  |
|  | Pre | CG (3) | 54.15 (7.76) |  |  |  |
|  | Post |  | 50.21 (5.63) |  |  |  |
| **R Knee Angle Max Swing** | Pre | EG (25) | 50.00 (10.40) | (-2.81, 7.38) | 0.364 | 0.19 |
|  | Post |  | 52.28 (14.68) |  |  |  |
|  | Pre | CG (3) | 53.26 (10.61) |  |  |  |
|  | Post |  | 55.19 (5.85) |  |  |  |
| **L Knee Angle Min Stance** | Pre | EG (25) | 1.04 (10.62) | (-1.29, 5.98) | 0.196 | 0.27 |
|  | Post |  | 3.38 (12.21) |  |  |  |
|  | Pre | CG (3) | 7.36 (4.61) |  |  |  |
|  | Post |  | 2.71 (6.12) |  |  |  |
| **R Knee Angle Min Stance** | Pre | EG (25) | 5.21 (15.40) | (-8.22, 6.26) | 0.783 | -0.06 |
|  | Post |  | 4.23 (8.18) |  |  |  |
|  | Pre | CG (3) | 1.51 (2.69) |  |  |  |
|  | Post |  | 1.43 (11.40) |  |  |  |
| **L Knee Angle Min Swing** | Pre | EG (25) | 2.54 (10.62) | (-1.79, 6.37) | 0.258 | 0.23 |
|  | Post |  | 4.83 (9.90) |  |  |  |
|  | Pre | CG (3) | 4.73 (8.58) |  |  |  |
|  | Post |  | 1.33 (8.60) |  |  |  |
| **R Knee Angle Min Swing** | Pre | EG (25) | 2.03 (9.01) | (-0.51, 7.56) | 0.084 | 0.36 |
|  | Post |  | 5.56 (8.54) |  |  |  |
|  | Pre | CG (3) | -0.89 (6.86) |  |  |  |
|  | Post |  | -1.46 (14.38) |  |  |  |

*Note: EG = Experimental Group (FVT); CG = Control Group (Sham). The p-value, 95% CI, and Effect Size (Cohen's d_z_) represent the within-group, pre-to-post-intervention change calculated exclusively for the EG.*

| **Appendix Table A3.** Kinetic Gait Variables (Pre- and Post-Intervention) | | | | | | |
| --- | --- | --- | --- | --- | --- | --- |
| **Outcome Measure** | **Time** | **Group (N)** | **Mean (SD)** | **EG 95% CI** | **EG p-Value** | **EG Effect Size (d)** |
| **Left Ankle Moment Max Stance** | Pre | EG (13) | 0.63 (0.49) | (-0.47, 0.25) | 0.515 | -0.19 |
|  | Post |  | 0.52 (0.47) |  |  |  |
|  | Pre | CG (1) | 1.23 (N/A) |  |  |  |
|  | Post |  | 1.02 (N/A) |  |  |  |
| **Left Ankle Moment Max Swing** | Pre | EG (10) | 0.03 (0.03) | (-0.04, 0.04) | 0.916 | 0.03 |
|  | Post |  | 0.03 (0.05) |  |  |  |
|  | Pre | CG (1) | 0.02 (N/A) |  |  |  |
|  | Post |  | 0.02 (N/A) |  |  |  |
| **Left Ankle Moment Min Stance** | Pre | EG (13) | -0.37 (0.46) | (-0.12, 0.06) | 0.492 | -0.20 |
|  | Post |  | -0.40 (0.48) |  |  |  |
|  | Pre | CG (2) | -0.04 (0.03) |  |  |  |
|  | Post |  | -0.13 (0.13) |  |  |  |
| **Left Ankle Moment Min Swing** | Pre | EG (13) | -0.02 (0.01) | (-0.01, 0.01) | 0.700 | -0.11 |
|  | Post |  | -0.02 (0.02) |  |  |  |
|  | Pre | CG (2) | -0.02 (0.00) |  |  |  |
|  | Post |  | -0.02 (0.00) |  |  |  |
| **Left Ankle Power Max Stance** | Pre | EG (13) | 1.31 (1.15) | (-0.51, 0.30) | 0.589 | -0.15 |
|  | Post |  | 1.20 (1.00) |  |  |  |
|  | Pre | CG (2) | 0.77 (1.08) |  |  |  |
|  | Post |  | 1.32 (0.54) |  |  |  |
| **Left Ankle Power Max Swing** | Pre | EG (13) | 0.10 (0.10) | (-0.15, 0.36) | 0.396 | 0.24 |
|  | Post |  | 0.20 (0.41) |  |  |  |
|  | Pre | CG (2) | 0.02 (0.00) |  |  |  |
|  | Post |  | 0.02 (0.01) |  |  |  |
| **Left Ankle Power Min Stance** | Pre | EG (13) | -1.40 (3.10) | (-0.98, 2.90) | 0.301 | 0.30 |
|  | Post |  | -0.43 (0.25) |  |  |  |
|  | Pre | CG (2) | -0.48 (0.64) |  |  |  |
|  | Post |  | -0.54 (0.02) |  |  |  |
| **Left Ankle Power Min Swing** | Pre | EG (13) | -0.07 (0.06) | (-0.21, 0.09) | 0.384 | -0.25 |
|  | Post |  | -0.14 (0.25) |  |  |  |
|  | Pre | CG (2) | -0.05 (0.01) |  |  |  |
|  | Post |  | -0.06 (0.07) |  |  |  |
| **Left Hip Moment Max Stance** | Pre | EG (12) | 1.11 (0.50) | (-0.57, 0.10) | 0.156 | -0.44 |
|  | Post |  | 0.88 (0.40) |  |  |  |
|  | Pre | CG (2) | 3.47 (4.02) |  |  |  |
|  | Post |  | 0.88 (0.10) |  |  |  |
| **Left Hip Moment Max Swing** | Pre | EG (12) | 0.16 (0.08) | (-0.06, 0.08) | 0.847 | 0.06 |
|  | Post |  | 0.17 (0.07) |  |  |  |
|  | Pre | CG (2) | 5.38 (7.43) |  |  |  |
|  | Post |  | 0.16 (0.14) |  |  |  |
| **Left Hip Moment Min Stance** | Pre | EG (12) | -0.29 (0.17) | (-0.20, 0.06) | 0.247 | -0.35 |
|  | Post |  | -0.36 (0.18) |  |  |  |
|  | Pre | CG (2) | -0.10 (0.44) |  |  |  |
|  | Post |  | -0.26 (0.11) |  |  |  |
| **Left Hip Moment Min Swing** | Pre | EG (11) | -0.40 (0.14) | (-0.15, 0.11) | 0.690 | -0.12 |
|  | Post |  | -0.42 (0.16) |  |  |  |
|  | Pre | CG (2) | -0.06 (0.57) |  |  |  |
|  | Post |  | -0.32 (0.05) |  |  |  |
| **Left Hip Power Max Stance** | Pre | EG (12) | 0.82 (0.51) | (-0.30, 0.09) | 0.243 | -0.36 |
|  | Post |  | 0.71 (0.40) |  |  |  |
|  | Pre | CG (2) | 0.09 (0.35) |  |  |  |
|  | Post |  | 0.54 (0.24) |  |  |  |
| **Left Hip Power Max Swing** | Pre | EG (12) | 0.39 (0.19) | (-0.10, 0.19) | 0.528 | 0.19 |
|  | Post |  | 0.44 (0.17) |  |  |  |
|  | Pre | CG (2) | -0.06 (0.47) |  |  |  |
|  | Post |  | 0.47 (0.06) |  |  |  |
| **Left Hip Power Min Stance** | Pre | EG (12) | -1.21 (0.91) | (-0.13, 0.77) | 0.144 | 0.45 |
|  | Post |  | -0.89 (0.63) |  |  |  |
|  | Pre | CG (2) | -0.23 (0.57) |  |  |  |
|  | Post |  | -0.76 (0.47) |  |  |  |
| **Left Hip Power Min Swing** | Pre | EG (12) | -0.20 (0.21) | (-0.08, 0.19) | 0.409 | 0.25 |
|  | Post |  | -0.14 (0.09) |  |  |  |
|  | Pre | CG (2) | 0.29 (0.61) |  |  |  |
|  | Post |  | -0.06 (0.01) |  |  |  |
| **Left Knee Moment Max Stance** | Pre | EG (13) | 0.66 (0.29) | (-0.19, 0.10) | 0.543 | -0.17 |
|  | Post |  | 0.62 (0.32) |  |  |  |
|  | Pre | CG (2) | 6.76 (8.99) |  |  |  |
|  | Post |  | 0.64 (0.01) |  |  |  |
| **Left Knee Moment Max Swing** | Pre | EG (13) | 0.09 (0.09) | (-0.08, 0.03) | 0.280 | -0.31 |
|  | Post |  | 0.06 (0.02) |  |  |  |
|  | Pre | CG (2) | 7.82 (11.02) |  |  |  |
|  | Post |  | 0.05 (0.05) |  |  |  |
| **Left Knee Moment Min Stance** | Pre | EG (13) | -0.16 (0.10) | (-0.07, 0.06) | 0.850 | -0.05 |
|  | Post |  | -0.16 (0.09) |  |  |  |
|  | Pre | CG (2) | -0.07 (0.13) |  |  |  |
|  | Post |  | -0.17 (0.10) |  |  |  |
| **Left Knee Moment Min Swing** | Pre | EG (13) | -0.26 (0.06) | (-0.05, 0.03) | 0.495 | -0.20 |
|  | Post |  | -0.27 (0.08) |  |  |  |
|  | Pre | CG (2) | -0.09 (0.25) |  |  |  |
|  | Post |  | -0.24 (0.09) |  |  |  |
| **Left Knee Power Max Stance** | Pre | EG (13) | 0.40 (0.33) | (-0.20, 0.25) | 0.790 | 0.08 |
|  | Post |  | 0.43 (0.21) |  |  |  |
|  | Pre | CG (2) | 0.06 (0.20) |  |  |  |
|  | Post |  | 0.29 (0.19) |  |  |  |
| **Left Knee Power Max Swing** | Pre | EG (13) | 0.14 (0.12) | (-0.05, 0.08) | 0.695 | 0.11 |
|  | Post |  | 0.15 (0.09) |  |  |  |
|  | Pre | CG (2) | 0.00 (0.27) |  |  |  |
|  | Post |  | 0.17 (0.22) |  |  |  |
| **Left Knee Power Min Stance** | Pre | EG (13) | -0.74 (0.47) | (-0.29, 0.22) | 0.769 | -0.08 |
|  | Post |  | -0.78 (0.61) |  |  |  |
|  | Pre | CG (2) | -0.10 (0.33) |  |  |  |
|  | Post |  | -0.80 (0.66) |  |  |  |
| **Left Knee Power Min Swing** | Pre | EG (12) | -0.88 (0.36) | (-0.30, 0.11) | 0.328 | -0.30 |
|  | Post |  | -0.97 (0.35) |  |  |  |
|  | Pre | CG (2) | -0.43 (0.65) |  |  |  |
|  | Post |  | -0.88 (0.63) |  |  |  |
| **Right Ankle Moment Max Stance** | Pre | EG (11) | 0.41 (0.47) | (-0.24, 0.54) | 0.400 | 0.26 |
|  | Post |  | 0.57 (0.46) |  |  |  |
|  | Pre | CG (2) | -2.48 (5.16) |  |  |  |
|  | Post |  | 1.01 (0.00) |  |  |  |
| **Right Ankle Moment Max Swing** | Pre | EG (12) | 0.02 (0.02) | (-0.02, 0.01) | 0.705 | -0.11 |
|  | Post |  | 0.02 (0.01) |  |  |  |
|  | Pre | CG (1) | -3.85 (N/A) |  |  |  |
|  | Post |  | 0.00 (N/A) |  |  |  |
| **Right Ankle Moment Min Stance** | Pre | EG (11) | -0.29 (0.52) | (-0.35, 0.07) | 0.162 | -0.46 |
|  | Post |  | -0.43 (0.48) |  |  |  |
|  | Pre | CG (2) | 0.41 (0.70) |  |  |  |
|  | Post |  | -0.10 (0.12) |  |  |  |
| **Right Ankle Moment Min Swing** | Pre | EG (12) | -0.02 (0.01) | (-0.01, 0.00) | 0.407 | -0.25 |
|  | Post |  | -0.02 (0.01) |  |  |  |
|  | Pre | CG (1) | 0.00 (N/A) |  |  |  |
|  | Post |  | -0.02 (N/A) |  |  |  |
| **Right Ankle Power Max Stance** | Pre | EG (11) | 0.98 (1.20) | (-0.43, 1.08) | 0.360 | 0.29 |
|  | Post |  | 1.30 (1.05) |  |  |  |
|  | Pre | CG (2) | 0.84 (1.34) |  |  |  |
|  | Post |  | 1.29 (0.47) |  |  |  |
| **Right Ankle Power Max Swing** | Pre | EG (12) | 0.04 (0.03) | (-0.01, 0.10) | 0.096 | 0.52 |
|  | Post |  | 0.08 (0.08) |  |  |  |
|  | Pre | CG (1) | -0.03 (N/A) |  |  |  |
|  | Post |  | 0.02 (N/A) |  |  |  |
| **Right Ankle Power Min Stance** | Pre | EG (11) | -0.40 (0.42) | (-0.40, 0.19) | 0.440 | -0.24 |
|  | Post |  | -0.50 (0.36) |  |  |  |
|  | Pre | CG (2) | 0.15 (1.60) |  |  |  |
|  | Post |  | -0.51 (0.17) |  |  |  |
| **Right Ankle Power Min Swing** | Pre | EG (12) | -0.07 (0.06) | (-0.04, 0.06) | 0.676 | 0.12 |
|  | Post |  | -0.07 (0.05) |  |  |  |
|  | Pre | CG (1) | 0.01 (N/A) |  |  |  |
|  | Post |  | -0.02 (N/A) |  |  |  |
| **Right Hip Moment Max Stance** | Pre | EG (10) | 0.82 (0.66) | (-0.45, 0.60) | 0.759 | 0.10 |
|  | Post |  | 0.89 (0.39) |  |  |  |
|  | Pre | CG (2) | 15.50 (21.22) |  |  |  |
|  | Post |  | 0.91 (0.02) |  |  |  |
| **Right Hip Moment Max Swing** | Pre | EG (11) | 0.14 (0.08) | (-0.02, 0.11) | 0.142 | 0.48 |
|  | Post |  | 0.19 (0.10) |  |  |  |
|  | Pre | CG (1) | 31.71 (N/A) |  |  |  |
|  | Post |  | 0.17 (N/A) |  |  |  |
| **Right Hip Moment Min Stance** | Pre | EG (10) | -0.29 (0.13) | (-0.23, 0.04) | 0.156 | -0.49 |
|  | Post |  | -0.38 (0.12) |  |  |  |
|  | Pre | CG (2) | 1.33 (2.39) |  |  |  |
|  | Post |  | -0.26 (0.07) |  |  |  |
| **Right Hip Moment Min Swing** | Pre | EG (11) | -0.39 (0.16) | (-0.14, 0.10) | 0.693 | -0.12 |
|  | Post |  | -0.41 (0.16) |  |  |  |
|  | Pre | CG (1) | 9.14 (N/A) |  |  |  |
|  | Post |  | -0.29 (N/A) |  |  |  |
| **Right Hip Power Max Stance** | Pre | EG (10) | 0.58 (0.37) | (-0.10, 0.36) | 0.237 | 0.40 |
|  | Post |  | 0.71 (0.39) |  |  |  |
|  | Pre | CG (2) | 0.66 (0.26) |  |  |  |
|  | Post |  | 0.65 (0.38) |  |  |  |
| **Right Hip Power Max Swing** | Pre | EG (11) | 0.38 (0.16) | (-0.39, 1.19) | 0.286 | 0.34 |
|  | Post |  | 0.78 (1.19) |  |  |  |
|  | Pre | CG (1) | 0.22 (N/A) |  |  |  |
|  | Post |  | 0.27 (N/A) |  |  |  |
| **Right Hip Power Min Stance** | Pre | EG (10) | -0.87 (0.76) | (-0.82, 0.53) | 0.637 | -0.15 |
|  | Post |  | -1.02 (0.60) |  |  |  |
|  | Pre | CG (2) | -0.15 (0.01) |  |  |  |
|  | Post |  | -0.82 (0.08) |  |  |  |
| **Right Hip Power Min Swing** | Pre | EG (11) | -0.17 (0.15) | (-1.45, 0.54) | 0.334 | -0.31 |
|  | Post |  | -0.62 (1.41) |  |  |  |
|  | Pre | CG (1) | -0.35 (N/A) |  |  |  |
|  | Post |  | 0.01 (N/A) |  |  |  |
| **Right Knee Moment Max Stance** | Pre | EG (11) | 0.50 (0.36) | (-0.15, 0.49) | 0.253 | 0.37 |
|  | Post |  | 0.68 (0.28) |  |  |  |
|  | Pre | CG (2) | 17.49 (24.55) |  |  |  |
|  | Post |  | 0.59 (0.09) |  |  |  |
| **Right Knee Moment Max Swing** | Pre | EG (12) | 0.05 (0.03) | (-0.00, 0.05) | 0.072 | 0.57 |
|  | Post |  | 0.07 (0.04) |  |  |  |
|  | Pre | CG (1) | 42.69 (N/A) |  |  |  |
|  | Post |  | 0.06 (N/A) |  |  |  |
| **Right Knee Moment Min Stance** | Pre | EG (11) | -0.15 (0.07) | (-0.08, 0.04) | 0.391 | -0.27 |
|  | Post |  | -0.17 (0.07) |  |  |  |
|  | Pre | CG (2) | 1.56 (2.60) |  |  |  |
|  | Post |  | -0.17 (0.07) |  |  |  |
| **Right Knee Moment Min Swing** | Pre | EG (12) | -0.24 (0.09) | (-0.08, 0.03) | 0.367 | -0.27 |
|  | Post |  | -0.27 (0.11) |  |  |  |
|  | Pre | CG (1) | 4.44 (N/A) |  |  |  |
|  | Post |  | -0.20 (N/A) |  |  |  |
| **Right Knee Power Max Stance** | Pre | EG (10) | 0.28 (0.33) | (-0.33, 0.23) | 0.713 | -0.12 |
|  | Post |  | 0.24 (0.20) |  |  |  |
|  | Pre | CG (2) | 0.33 (0.23) |  |  |  |
|  | Post |  | 0.19 (0.20) |  |  |  |
| **Right Knee Power Max Swing** | Pre | EG (12) | 0.14 (0.12) | (-0.04, 0.11) | 0.328 | 0.30 |
|  | Post |  | 0.18 (0.14) |  |  |  |
|  | Pre | CG (1) | 0.07 (N/A) |  |  |  |
|  | Post |  | 0.03 (N/A) |  |  |  |
| **Right Knee Power Min Stance** | Pre | EG (11) | -0.57 (0.51) | (-0.70, 0.19) | 0.234 | -0.38 |
|  | Post |  | -0.83 (0.64) |  |  |  |
|  | Pre | CG (2) | -0.26 (0.31) |  |  |  |
|  | Post |  | -0.89 (0.62) |  |  |  |
| **Right Knee Power Min Swing** | Pre | EG (9) | -0.85 (0.47) | (-0.24, 0.26) | 0.950 | 0.02 |
|  | Post |  | -0.85 (0.42) |  |  |  |
|  | Pre | CG (0) | N/A |  |  |  |
|  | Post |  | N/A |  |  |  |
| **Right GRF RHS** | Pre | EG (7) | 0.00 (0.00) | (0.17, 0.42) | **0.001*** | 2.17 |
|  | Post |  | 0.29 (0.14) |  |  |  |
|  | Pre | CG (2) | 0.00 (0.00) |  |  |  |
|  | Post |  | 0.41 (0.30) |  |  |  |
| **Right GRF RTO** | Pre | EG (6) | 0.20 (0.35) | (-0.48, 0.47) | 0.984 | -0.01 |
|  | Post |  | 0.20 (0.16) |  |  |  |
|  | Pre | CG (2) | 0.15 (0.21) |  |  |  |
|  | Post |  | 0.18 (0.26) |  |  |  |
| **Left GRF LHS** | Pre | EG (7) | 0.00 (0.00) | (-0.16, 1.00) | 0.126 | 0.67 |
|  | Post |  | 0.42 (0.63) |  |  |  |
|  | Pre | CG (2) | 0.00 (0.00) |  |  |  |
|  | Post |  | 0.50 (0.34) |  |  |  |
| **Left GRF LTO** | Pre | EG (7) | 0.00 (0.00) | (0.01, 0.32) | **0.041*** | 0.98 |
|  | Post |  | 0.16 (0.17) |  |  |  |
|  | Pre | CG (2) | 4.50 (6.36) |  |  |  |
|  | Post |  | 0.09 (0.13) |  |  |  |

*Note: EG = Experimental Group (FVT); CG = Control Group (Sham). The p-value, 95% CI, and Effect Size (Cohen's d_z_) represent the within-group, pre-to-post-intervention change calculated exclusively for the EG. Missing standard deviations (N/A) occur when a group's N = 1, precluding variance calculations.*
